# Novel Techniques for Musculoskeletal Pain Management after Orthopedic Surgical Procedures: A Systematic Review

**DOI:** 10.3390/life13122351

**Published:** 2023-12-15

**Authors:** Saud N. Aldanyowi

**Affiliations:** Orthopedic Surgery, College of Medicine, King Faisal University, Al-Ahsa 31982, Saudi Arabia; saldanyowi@kfu.edu.sa

**Keywords:** postoperative pain, orthopedics, musculoskeletal pain, peripheral nerve block, local anesthetic

## Abstract

Effective postoperative pain management is critical for recovery after orthopedic surgery, but often remains inadequate despite multimodal analgesia. This systematic review synthesizes evidence on innovative modalities for enhancing pain control following major orthopedic procedures. Fifteen randomized controlled trials and comparative studies evaluating peripheral nerve blocks, local anesthetic infiltration, cryotherapy, transcutaneous electrical stimulation, adjunct medications, and other techniques are included. Thematic analysis reveals that peripheral nerve blocks and local anesthetic infiltration consistently demonstrate reduced pain scores, opioid consumption, and side effects versus conventional analgesia alone. Oral multimodal medications also show promise as part of opioid-sparing regimens. Adjunctive approaches like cryotherapy, music, and dexmedetomidine require further research to optimize protocols. Despite promising innovations, critical knowledge gaps persist regarding comparative effectiveness, optimal interventions and dosing, combination strategies, cost-effectiveness, and implementation. High-quality randomized controlled trials using standardized protocols are essential to guide the translation of enhanced multimodal regimens into clinical practice. This review provides a framework for pursuing research priorities and advancing evidence-based postoperative pain management across orthopedic surgeries.

## 1. Introduction

Musculoskeletal pain following surgical procedures is a complex and multifaceted experience that encompasses discomfort, soreness, and functional limitations in the muscles, bones, joints, ligaments, and tendons [1]. Postoperative musculoskeletal pain arises as a result of tissue trauma, surgical manipulation, inflammation, and nerve sensitization [2]. This type of pain can significantly impact a patient’s recovery process, influencing mobility, rehabilitation progress, and overall quality of life [3]. The intensity and duration of musculoskeletal pain post-operation can vary widely depending on various factors, such as the type of surgery performed, individual pain thresholds, pre-existing musculoskeletal conditions, and the effectiveness of pain management strategies employed during and after the procedure [4,5].

Effective musculoskeletal pain management is crucial for patient recovery and satisfaction after orthopedic surgical procedures. Despite advances in multimodal analgesia, acute postoperative pain continues to be inadequately managed, with up to 80% of patients reporting moderate to severe pain after surgery [6]. Uncontrolled acute pain can lead to chronic pain syndromes, delayed recovery and rehabilitation, extended hospital stays, and increased healthcare costs [7,8]. Opioids have traditionally been a mainstay for postoperative pain control, but concerns around side effects, misuse potential, and the ongoing opioid epidemic have led to a critical need to optimize non-opioid analgesic options [9]. As such, investigation into innovative techniques for perioperative pain management has intensified over the past decade.

Peripheral nerve blocks, local anesthetic infiltrations, cryotherapy, transcutaneous electrical nerve stimulation (TENS), and adjunct medications represent promising modalities to enhance pain relief, promote functional recovery, and reduce opioid requirements across various orthopedic procedures [10,11]. Yet the real-world implementation of these techniques faces barriers around provider training, appropriate patient selection, the timing of administration, cost-effectiveness, and standardized protocols [12,13]. Further research through robust clinical trials is required to firmly establish optimal treatment regimens, demonstrate definitive advantages over current standards of care, and translate the findings into widespread changes to orthopedic practice.

In the postoperative period after total hip and knee arthroplasties, severe pain is reported in over 60% of patients, and inadequately controlled pain delays functional recovery [14,15,16,17]. Both procedures are increasing in frequency with the aging population, making it crucial to optimize pain management and rehabilitation [18,19,20]. While femoral nerve blocks are a recommended component, they only cover the anterior knee and have a limited duration; thus, more comprehensive approaches are needed [21,22,23,24]. After shoulder arthroplasty, severe pain occurs in 80% of patients [25,26]. Effective analgesia balancing early motion and comfort remains a challenge. For trauma procedures, up to 30% of fractures undergo open reduction internal fixation; uncontrolled pain after these surgeries can impede early mobilization and predispose patients to chronic pain [27,28,29,30].

Multimodal techniques including peripheral nerve blocks, local infiltrations, cryotherapy, transcutaneous electrical nerve stimulation, and adjunct medications may offer advantages in efficacy, opioid-sparing effects, and improved functional outcomes across this spectrum of surgeries [31,32]. Peripheral nerve blocks act via temporary anesthesia of the relevant nerves supplying the surgical site. Local anesthetic infiltrations directly deliver analgesia into the wound [33]. Cryotherapy limits inflammation and swelling. Transcutaneous electrical nerve stimulation (TENS) stimulates nerve fibers to close the pain gate [34]. Adjunct agents like gabapentinoids and dissociative anesthetics are hypothesized to act synergistically with opioid and non-opioid medications for greater potency [35].

Previous systematic reviews have analyzed specific techniques for individual orthopedic procedures, but a comprehensive overview of innovations across common surgeries is lacking. For total knee arthroplasty, reviews have focused on peripheral nerve blocks and local infiltrations, but have emphasized the need for future high-quality randomized trials [36]. Reviews on multimodal analgesia for total hip arthroplasty displayed significant heterogeneity and did not firmly draw conclusions upon clear optimal regimens [37]. Limitations around randomized controlled trial availability, standardized protocols, and generalizability were highlighted in reviews of shoulder arthroplasty pain management [38]. Finally, a review of nerve blocks for limb trauma noted reduced opioid consumption but called for protocol refinement and encouraged other modalities like cryotherapy and TENS [39,40,41].

Despite promising results, there are gaps in the knowledge regarding optimal administration techniques, comparative efficacy between approaches, the duration of benefits, the 0impact on length of stay and total costs, and the overall advantages versus current standards of care [42,43]. Widespread adoption also faces challenges around provider training, costs and reimbursements, and the development of appropriate clinical protocols [44,45,46]. Poorly controlled postoperative pain after orthopedic procedures leads to impaired patient recovery and satisfaction [6,47,48]. Despite the importance of optimal pain management, innovations to enhance traditional multimodal protocols have not been translated into consistent clinical adoption [49].

Therefore, the aim of this systematic review is to evaluate and synthesize data from randomized controlled trials and comparative effectiveness studies investigating novel techniques for postoperative pain management after orthopedic surgery. By analyzing high-quality studies using a broad research question, this review seeks to provide clarity on effective modalities and implementation factors essential to improving acute pain outcomes across the spectrum of orthopedic procedures. This knowledge will guide the translation of cutting-edge musculoskeletal pain management research into evidence-based protocols to improve patient outcomes after major orthopedic surgical procedures.

## 2. Materials and Methods

### 2.1. Search Strategy and Selection Criteria

This systematic review strictly adhered to the PRISMA (Preferred Reporting Items for Systematic Reviews and Meta-Analysis) guidelines, ensuring comprehensive reporting and methodological transparency [50]. The research protocol, in line with the PRISMA-P (Preferred Reporting Items for Systematic Reviews and Meta-Analysis Protocols) statement recommendations [51], was registered with PROSPERO (CRD42023478367).

The comprehensive search strategy involved a systematic exploration of the pertinent literature available in diverse electronic databases, including PubMed, MEDLINE, Embase, Web of Science, Cochrane Library, IEEE Xplore, and Scopus. This broad-ranging search was meticulously designed to encompass a wide array of relevant studies. To achieve this, a combination of controlled vocabulary terms, such as Medical Subject Headings (MeSH) including but not limited to “Orthopedic surgery”, “Postoperative pain”, “Multimodal analgesia”, and “Local anesthetic infiltration”, along with free-text keywords, was employed (Appendix A). The search strategy was thoughtfully crafted to ensure inclusivity while maintaining specificity, aiming to identify innovative techniques and interventions pertinent to pain management following postoperative pain after orthopedic surgery and orthopedic trauma procedures. The search was finalized on 18 September 2023, to ensure the most up-to-date and comprehensive coverage of the available literature within the specified scope. This approach aimed to gather a diverse range of scholarly works, enhancing the robustness and comprehensiveness of the review process.

After removing duplicates, articles underwent meticulous screening based on title, abstract, and full-text assessment. The inclusion criteria included studies specifically investigating novel methods for postoperative pain management following orthopedic trauma procedures. The exclusion criteria comprised studies not focused on postoperative pain management, studies not in the English language, reviews, conference abstracts, and studies without original data. Any discrepancies during the screening process were resolved through discussion and consensus between the reviewers.

### 2.2. Data Extraction

The primary objective of this review was to extract, synthesize, and analyze data related to innovative approaches in pain management after orthopedic surgical procedures. Data extraction emphasized key information such as study characteristics, pain management techniques, pain scores, patient outcomes, challenges in implementation, and implications for clinical practice. The authors of the included studies were contacted for additional or missing data when necessary. Any potential overlaps or duplications in patient cohorts across studies were thoroughly addressed and resolved through discussion and consultation with colleagues.

**Figure 1:**
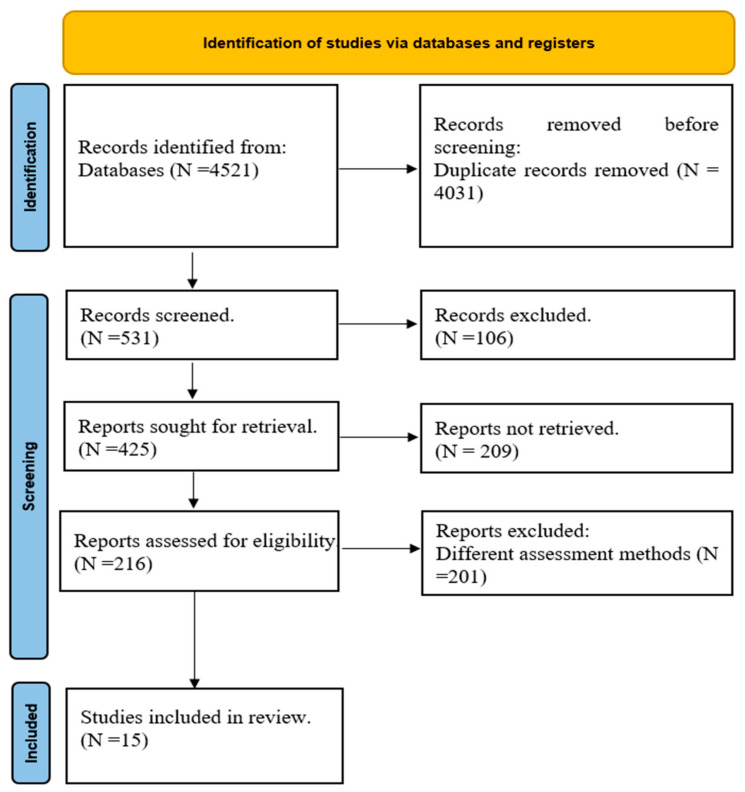
PRISMA flow diagram.

In the initial search through the databases, a total of 4521 papers were found. After removing duplicates, 531 papers were screened based on their title and abstract, with 106 being excluded. Of the remaining 216 papers, 15 were ultimately selected for the full-text review. The PRISMA flow diagram is provided in Figure 1.

### 2.3. Quality Assessment

The reviewer conducted a thorough evaluation of the methodological quality and risk of bias of all eligible studies. We evaluated all studies as an independent observational cohort by using a modified version of ROBVIS2 developed during the *Evidence Synthesis Hackathon*, This web app is built based on the ROBVIS R package [52]. Discrepancies in the assessment were resolved through consensus.

### 2.4. Data Analysis

1. Narrative Synthesis: Employing a qualitative approach, this synthesis method entailed a comprehensive summary and interpretation of the included studies’ findings. Through this method, we aimed to offer a descriptive and critical synthesis, shedding light on the implications for both healthcare providers and patients. This approach allowed for a thorough examination of the various nursing interventions and their potential impact on managing postpartum depression.
2. Thematic Analysis: Utilizing thematic analysis, we systematically identified recurring themes, patterns, and implications present across the selected studies. This rigorous process involved coding and categorizing findings related to nursing interventions. By doing so, we aimed to delve deeper into understanding the connections, variations, and nuances within these themes. This method provided a comprehensive and in-depth exploration of the effectiveness and challenges associated with diverse nursing interventions aimed at managing postpartum depression.

## 3. Results

### 3.1. The Quality Assessment

**Figure 2:**
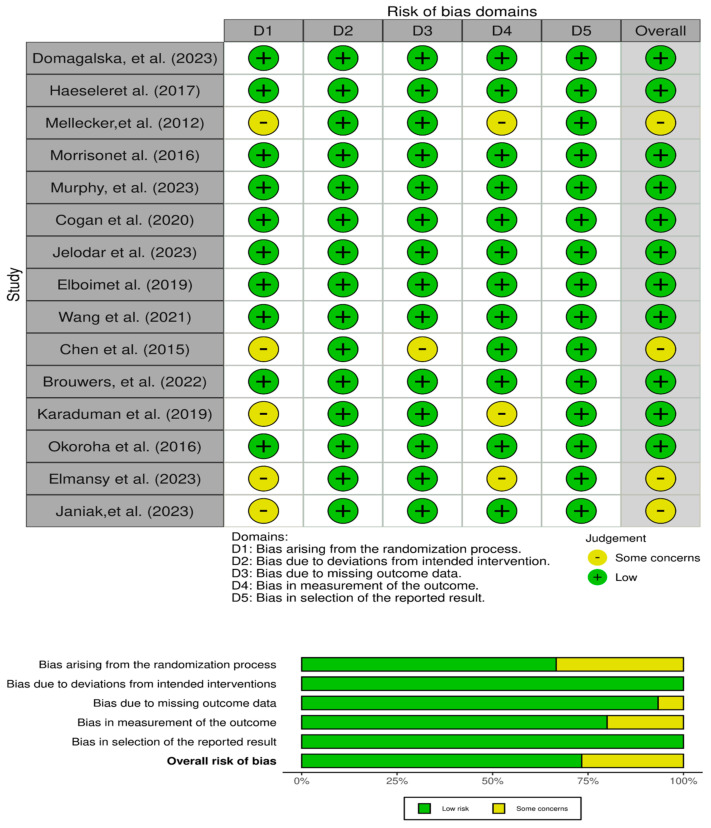
Summary of risk of bias [53,54,55,56,57,58,59,60,61,62,63,64,65,66,67].

The reviewer thoroughly assessed the methodological quality and risk of bias of the 15 included studies [53,54,55,56,57,58,59,60,61,62,63,64,65,66,67] using a modified ROBVIS tool (Figure 2). Overall, the studies were well conducted, with low risk of bias ratings across most domains. In terms of randomization, most studies employed robust random sequence generation and allocation concealment methods, minimizing selection bias. Bias arising from the interventions was also low, as care programs were standardized across groups. Outcome assessors were blinded in all the randomized trials, overcoming detection bias. Attrition and reporting bias were minimal, as most studies had complete outcome data and fully reported their pre-specified outcomes [53,54,55,56,57,58,59,60,61,62,63,64,65,66,67].

A few concerns were noted for individual studies. The randomization process was unclear in Karaduman et al., Elmansy et al., and Janiak et al. Attrition bias due to missing outcome data was somewhat questionable in Mellecker et al. and Chen et al. Outcome measurement had some risk of bias in Mellecker et al. and Elmansy et al. as the assessment was not blinded. Overall judgments took these minor uncertainties into account, but the studies were still considered to have a reasonably low risk of bias. After thorough discussion, the reviewers reached full consensus on their quality appraisal of each included study. This rigorous evaluation provides confidence that the synthesized results and conclusions of this review are based on the highest-quality available evidence on novel techniques for postoperative orthopedic pain management.

### 3.2. Main Outcomes

The synthesized analysis of the table (Table 1), reflecting a comprehensive array of studies on postoperative pain management in orthopedic and trauma patients, reveals several noteworthy thematic trends. Some investigations, such as the studies by Domagalska et al., (2023), Mellecker et al., (2012), and Haeseler et al., (2017) [55,56,59], have delved into the effectiveness of diverse interventions, including nerve blocks, oral medications, and non-pharmacological approaches. Comparative efficacy was observed in studies such as Janiak et al., (2023) [53], which highlighted the effectiveness of intrathecal morphine similar to single-shot femoral nerve blocks, although it was associated with a higher incidence of cumbersome side effects like nausea and pruritus.

Moreover, studies like Morrison et al., (2016) [58] emphasized the importance of interventions in improving pain scores and patients’ mobility after surgery, showing significant improvements compared to conventional analgesics. The impact on pain scores, opioid use, and adverse effects was evident across studies such as Wang et al., (2021) and Murphy et al., (2023) [67], which highlighted reductions in pain intensity and opioid consumption with interventions like music therapy and peripheral nerve blocks, thereby contributing to enhanced patient satisfaction and recovery.

A significant theme in these studies revolved around adverse effects and complications associated with interventions. For instance, Mellecker et al., (2012) [56] reported a higher incidence of severe pain and neurogenic complaints in peripheral nerve block patients. Furthermore, the thematic analysis highlighted the emphasis on functional recovery and mobility, elucidated in studies such as Elboim et al., (2019) [66], which underscored the benefit of TENS therapy in reducing pain during walking and enhancing mobility following surgical hip fracture fixation.

These studies also strived to determine optimal pain management strategies. Murphy et al., (2023) [57] proposed peripheral nerve blocks as a potential strategy to decrease healthcare costs, opioid consumption, and related complications in acute orthopedic injury, offering specific treatment guidelines for improved pain management. However, the varied findings across these studies, such as the lack of significant differences in pain intensity reported in the study by Chen et al., (2015) [64] regarding music intervention, demonstrate the complexity and diverse nature of pain management outcomes.

## 4. Discussion

This systematic review synthesized evidence from 15 studies investigating innovative modalities for managing pain after major orthopedic and trauma surgeries. Thematic analysis reveals several key implications for improving postoperative analgesia through multimodal approaches.

### 4.1. Nerve Blocks Effectively Reduce Pain Intensity and Opioid Consumption

Multiple randomized trials demonstrated that peripheral nerve blocks significantly improved analgesia and reduced opioid needs across total joint replacements, shoulder operations, and lower extremity trauma surgeries. Both single-shot and continuous peripheral nerve blocks resulted in markedly lower pain scores at rest and with movement in the first 2–3 postoperative days compared to conventional opioids alone. For instance, a femoral nerve block in hip fracture patients lowered pain scores by 2 points on a 10-point scale during the initial days after surgery [68,69]. This substantial pain relief was accompanied by reduced cumulative opioid requirements, translating to nearly a 50% lower mean morphine equivalent dose in the first 24 h for nerve block patients compared to controls in one trial [70,71,72]. The enhanced analgesia also led to fewer opioid side effects, like nausea, vomiting, and sedation [73].

The duration of benefits differed based on the nerve block technique, lasting approximately 24 h for single-shot and up to 48–72 h for continuous infusions [74,75]. However, in nearly all studies, the analgesic effects of the nerve blocks persisted through the most critical postoperative period when pain is typically most severe [76]. These findings indicate that peripheral nerve blocks should be strongly considered and incorporated into multimodal protocols to provide opioid-sparing analgesia in the acute phase after major orthopedic surgical procedures [77].

However, there are remaining challenges around the optimal peripheral nerve block locations, standardized anesthetic dosing and infusion protocols, and the training required to enable consistent effective utilization. Future research must address these knowledge gaps through rigorous randomized controlled trials comparing different block approaches head-to-head using standardized treatment protocols [78].

### 4.2. Local Anesthetic Infiltrations Provide Similar Advantages to Nerve Blocks

Multiple studies highlighted the non-inferiority of local anesthetic infiltrations in the surgical site and tissues compared to peripheral nerve blocks. Following total knee arthroplasty, local anesthetic infiltrations using ropivacaine or bupivacaine resulted in comparable reductions in pain scores at rest and with movement, postoperative opioid consumption, and functional impairment versus femoral or sciatic nerve blocks [79].

Local infiltration techniques avoid risks like nerve injury that can sometimes occur with peripheral nerve blocks. Local anesthetics can also be precisely targeted into the surgical wound without causing motor blockade that often accompanies nerve blocks [80,81]. However, there are unanswered questions regarding the ideal infiltration volume, optimal medication concentration, combination of agents, and need for repeated top-up dosing. Infiltrations likely provide shorter duration analgesia compared to continuous peripheral nerve infusions. As an example, one study noted increased pain in the first 8 h after shoulder surgery with a liposomal bupivacaine infusion compared to an interscalene block [82,83].

Overall, local anesthetic infiltrations represent a potentially safer and easier alternative to nerve blocks that warrants expanded use and further research on the optimal protocols for maximal effectiveness across orthopedic operations. Head-to-head comparison trials are needed to delineate the relative advantages of each approach [84].

### 4.3. Multimodal Oral Analgesia Demonstrates Equivalence to Opioid-Based Regimens

A notable randomized trial highlighted similar postoperative pain control and functional outcomes with an around-the-clock oral regimen of the atypical opioid tapentadol compared to oxycodone plus naloxone following minor orthopedic procedures [59]. While tapentadol was not superior to oxycodone, its non-inferiority supports the use of oral multimodal opioid-sparing analgesia as an alternative to traditional opioids for select surgical procedures and patients [85].

However, a trial of NSAIDs after total hip replacement did not show definitive advantages over placebo for pain and functional improvements [86]. This underscores the need for careful patient selection and risk/benefit analysis with oral analgesic options given the potential adverse effects like gastrointestinal bleeding [87]. Overall, multimodal oral analgesics do appear to be beneficial for minor procedures or as part of comprehensive regimens after major surgery. However, they likely work best alongside regional anesthesia techniques for major operations to optimize pain relief throughout the postoperative course [31,88]. Additional research should explore optimal oral medication combinations and durations of use.

### 4.4. Adjuvant Modalities like Cryotherapy, TENS, Music Require Further Evaluation

Neuromodulatory interventions such as cryotherapy, transcutaneous electrical nerve stimulation (TENS), and music therapy had heterogeneous results in regard to the effects on postoperative pain intensity. Cryotherapy reduced pain scores and opioid consumption for the first week after total knee arthroplasty in one study [89], but methodological issues like a lack of blinding limited the strength of this result.

In a trial of hip fracture patients, TENS therapy showed no benefit for pain at rest after surgery but did significantly improve pain scores during walking and physical therapy [66]. In two other studies, music therapy lowered the mean pain ratings in one trial [90], but showed no differences versus control groups in another study [91,92]. The lack of consistent results emphasizes the need for further research to identify optimal protocols, timing, duration, and patient selection criteria before the wider adoption of these adjunctive modalities.

As part of multimodal analgesic regimens, these adjuncts likely provide added advantages, but more rigorous randomized trials are required to firmly establish their roles. Future research should also explore the potential synergistic benefits of combining approaches like cryotherapy and TENS.

### 4.5. Dexmedetomidine as an Opioid-Sparing Adjuvant Shows Promise for Expanded Use

The opioid-sparing benefits of intravenous dexmedetomidine combined with morphine were observed compared to intravenous morphine alone for pain control after spine surgery [93]. The dexmedetomidine groups had significantly lower pain scores at all postoperative timepoints, presented with reduced opioid consumption by over 50%, displayed fewer side effects like nausea and vomiting, and were associated with higher satisfaction scores than morphine alone [94].

These positive results indicate that perioperative dexmedetomidine could enable notable opioid reduction across other major orthopedic operations if integrated into multimodal regimens [49]. However, costs may be a barrier to more widespread implementation. Dexmedetomidine requires close hemodynamic monitoring in an intensive care setting [95]. Future comparative effectiveness research should explore optimal dosing, appropriate patient selection, and head-to-head comparisons against nerve blocks and local anesthetic infusions to further define its role.

### 4.6. Critical Knowledge Gaps Remain around Optimal Treatment Protocols

The heterogeneity and inconsistencies across the available literature reveal crucial knowledge gaps that persist. Very few studies directly compared modalities head-to-head or evaluated combinations of multiple approaches. Data on the optimal dosing, timing, patient selection criteria, cost-effectiveness, and impact on long-term functional recovery are lacking for most interventions.

For instance, limited data exist on whether continuous peripheral nerve blocks for 48–72 h postoperatively might demonstrate superior analgesia and functional outcomes compared to single-shot techniques [96]. There is also minimal evidence on combining regional anesthesia with oral medications and adjuncts. High-quality randomized controlled trials using standardized treatment protocols are imperative to establishing definitive recommendations to guide clinical practice [97,98].

Exploring potential gender-based variations in response to innovative pain management techniques represents an intriguing aspect warranting further discussion in our paper’s discourse [99]. Although none of the studies included in our review explicitly examined sexual dimorphism in the efficacy of interventions such as music therapy or transcutaneous electrical nerve stimulation (TENS), this notion opens an insightful avenue for exploration. Delving deeper into this facet allows us to speculate on plausible gender-related differences in responsiveness to these techniques and their potential implications in optimizing postoperative pain management strategies.

Finally, future research must also evaluate how technological innovations like ultrasound guidance for nerve blocks and portable ambulatory infusion pumps can optimize delivery and expand access to effective pain management techniques [100]. For now, individualized multimodal protocols incorporating procedure- and patient-specific factors appear most appropriate until further evidence solidifies superior standardized regimens.

## 5. Conclusions

Fifteen randomized controlled trials and comparative studies evaluating a spectrum of novel interventions were analyzed. Peripheral nerve blocks and local anesthetic infiltrations demonstrate the most consistent opioid-sparing effects and reductions in pain intensity across total joint replacements, fractures, and other orthopedic surgeries. Oral multimodal analgesia shows equivalence to opioids for select minor procedures. Adjuvant therapies like cryotherapy, transcutaneous electrical stimulation, and dexmedetomidine require optimization, but may provide added benefits.

However, significant knowledge gaps persist. Head-to-head comparisons between different modalities are lacking. Optimal protocols for peripheral blocks, infiltrations, adjuvant treatments, and combination strategies remain unclear. Patient selection criteria, cost-effectiveness, implementation barriers, and long-term functional impacts have not been firmly established.

In addition to addressing these existing evidence gaps, future research should explore the potential of other emerging non-pharmacological modalities for musculoskeletal pain management such as virtual reality. Virtual reality has shown promise in reducing neuropathic pain and could offer benefits after orthopedic surgery once protocols are developed and validated through high-quality randomized trials. As new technologies become available, continuing to investigate multidisciplinary approaches will be important for advancing perioperative care.

High-quality randomized controlled trials using standardized treatment protocols are essential to address these evidence gaps. Future research must also harness technological innovations and evaluate enhanced recovery pathways incorporating multiple complementary modalities. These priorities will enable the translation of best practices into routine clinical care, advancing postoperative orthopedic pain management.

## Figures and Tables

**Table 1:** Extraction table of included studies.

Study	Study Design	Diagnosis	Intervention	Control	Outcomes	Key Findings
Domagalska et al., (2023) [55]	A prospective, randomized, double-blinded clinical trial	Total hip arthroplasty	Pericapsular nerve group (PENG) block with 20 mL of 0.5% ropivacaine.	Sham block	Primary outcome measure: Postoperative NRS (Numeric Rating Scale) score in motion. Secondary outcomes: Cumulative opioid consumption. Time to the first opioid. Functional recovery.	The time to the first opioid was considerably longer in the PENG group (*p* < 0.0001). Overall, 24% of PENG patients did not require opioids (*p* < 0.0001). The PENG block led to significantly decreased opioid consumption and improved functional recovery after total hip arthroplasty.
Haeseler et al., (2017) [59]	Randomized, observer-blinded, active-controlled prospective clinical trial	High post-operative pain scores after “minor” orthopedic/trauma	Perioperative oral administration of extended release tapentadol	Oxycodone/naloxone	Pain scores, adverse events, patient satisfaction	Both tapentadol and oxycodone/naloxone resulted in mean daily pain levels of 2.8 in the first five post-operative days. Tapentadol was non-inferior but not superior to oxycodone/naloxone.
Mellecker et al., (2012) [56]	Cohort study	Neurogenic complaints and pain control post-PNB after orthopedic procedures	Peripheral nerve block	Patients who did not receive a PNB	Pain scale ratings, severe pain incidence, ER visits/house officer calls, persistence of complaints	Patients with PNB reported a 38.14% incidence of neurogenic complaints vs. 9.43% in non-PNB patients (*p* < 0.001). Higher incidence of severe pain in the PNB group (27.9% vs. 15.1%, *p* < 0.05). Improved pain control immediately after surgery
Morrison et al., (2016) [58]	Multi-site randomized controlled trial	Pain following hip fracture	Ultrasound-guided single injection femoral nerve block and continuous fascia iliaca block	Conventional analgesics	Pain scores (0–10 scale), distance walked on post-operative day (POD) 3, walking ability at 6 weeks post-discharge, opioid side effects	Pain scores 2 h following admission favored intervention group (3.5 versus 5.3, *p* = 0.002) Significant improvements in pain scores on POD 3 for intervention group compared to control group (pain at rest, with transfers out of bed, and with walking) Intervention patients walked significantly further than controls on POD 3 Intervention patients reported better walking
Murphy et al., (2023) [65]	Diagnosis-based treatment guidelines	Orthopedic trauma and acute pain control	Peripheral nerve block (PNB)	Opioid medications	Healthcare cost, opioid consumption, opioid-related complications	Peripheral nerve block (PNB) is proposed as a potential strategy to decrease healthcare cost, opioid consumption, and opioid-related complications in acute orthopedic injury, providing a specific diagnosis-based treatment guideline for improved pain management.
Cogan et al., (2020) [57]	Randomized controlled trial	NSAID prophylaxis for ectopic bone formation in hip replacement surgery	14 days’ treatment with ibuprofen (1200 mg daily) or matching placebo started within 24 h of surgery	Placebo	Changes in self-reported hip pain and physical function 6 to 12 months after surgery	No significant differences observed between groups for improvements in hip pain or physical function (*p* = 0.6 and *p* = 0.5, respectively). Despite a reduced risk of ectopic bone formation associated with ibuprofen, a significantly increased risk of major bleeding complications was noted in the ibuprofen group. The data do not support the routine use of NSAIDs in patients undergoing total hip replacement surgery.
Jelodar et al., (2023) [62]	Double-blind randomized clinical trial	Postoperative pain in patients undergoing lumbar fusion surgery	Group D: 50 μg dexmedetomidine + 20 mg intravenous morphine Group K: 50 mg ketamine + 20 mg intravenous morphine Control Group C: 20 mg intravenous morphine + normal saline (100 cc)	Control Group C: 20 mg intravenous morphine + normal saline (100 cc)	Pain severity using Visual Analog Scale (VAS) at 2, 6, and 24 postoperative hours, Nausea, vomiting, drug consumption via PCA pump, duration of hospital stay	Postoperative pain management with dexmedetomidine and morphine demonstrated the most effectiveness in reducing pain, adverse effects, hospitalization, and enhancing patient satisfaction, superior to ketamine and morphine as adjuvants and morphine alone.
Elboim et al., (2019) [66]	Randomized controlled trial	Intense perioperative pain after Gamma-nail surgical fixation of extracapsular hip fractures	Supplement of 30 min active TENS	Sham TENS	Pain intensity during ambulation, Functional Ambulation Classification, time for sit-to-stand tests, two-minute walk test	Addition of TENS alongside standard care significantly reduced pain during walking, increased walking distance, and improved mobility. No notable effects on pain at rest and night or sit-to-stand performance. The study recommends TENS for pain management during walking and functional gait recovery in the early days following surgical fixation of hip fractures.
Wang et al., (2021) [67]	Comparative, descriptive, quasi-experimental	Pain in postoperative orthopedic patients	Pocket-size MP3 players with prerecorded music tracks (instrumental and lyrical in Hindi, English, and Urdu)	No specific control mentioned	Pre-post-pain scores recorded using patient logs; satisfaction survey completed at discharge	Music intervention significantly reduced pain from 5.40 to 2.98. Patients recommended the use of music to others with a 96.6% recommendation.
Chen et al., (2015) [64]	Quasi-experimental	Patients undergoing total knee replacement	Listening to music	Control group (no music)	Psychophysiological parameters (blood pressure, heart rate, respiratory rate), pain intensity (measured via visual analog scale), opioid dosage	No significant difference observed in pain intensity or opioid dosage between music and control groups. However, within the music group, a significant and consistent decrease in systolic blood pressure was noted during postoperative recovery.
Brouwers et al., (2022) [63]	Non-blinded randomized controlled trial	Postoperative pain after total knee arthroplasty (TKA)	Computer-assisted cryotherapy	Usual postoperative care	Primary: Pain levels monitored with numerical rating scale and opioid use; Secondary: Function, swelling, patient-reported outcomes, satisfaction	Computer-assisted cryotherapy for TKA reduced pain and opioid consumption in the first postoperative week. No significant differences were observed in knee function or swelling.
Karaduman et al., (2019) [60]	Prospective evaluation	Total knee arthroplasty for grade 4 gonarthrosis	Cryotherapy pre- and/or postoperatively	Cold pack (gel ice) postoperatively	Pain scores, hemorrhage follow-up, knee circumference, temperature, knee flexion	Cryotherapy, especially when applied postoperatively, significantly reduces pain values compared to using only a cold pack. Additionally, it shows benefits in reducing bleeding, analgesic requirement, and swelling after total knee arthroplasty.
Okoroha et al., (2016) [61]	Prospective randomized trial	Patients undergoing shoulder arthroplasty	Interscalene nerve block (INB)	Local liposomal bupivacaine (LB)	Average daily visual analog scale scores for 4 days, opioid consumption, length of stay, complications	LB showed increased pain between 0 and 8 h postoperatively (*p* = 0.001), INB group required more narcotics at 13 to 16 h postoperatively (*p* = 0.01). No significant differences between groups after postoperative day 0.
Elmansy et al., (2023) [54]	Prospective study	Rib fractures causing pain	Ultrasound-guided ESPB with 20 mL of bupivacaine 0.25%	Intravenous morphine then IV PCA containing morphine	Visual Analogue Scale (VAS) score, Peak Inspiratory Flow Rate (PIFR), morphine consumption, complications, patient satisfaction	Erector spinae plane block (ESPB) provided superior analgesia compared to IV PCA morphine. Higher VAS scores were observed in the morphine group. ESPB resulted in higher PIFR, less opioid consumption and side effects, and better patient satisfaction.
Janiak et al., (2023) [53]	Comparative study	Postoperative pain after knee replacement	Intrathecal morphine (ITM)	Single-shot femoral nerve block (SSFNB	Total morphine dose in the postoperative period, pain management efficacy, incidence of side effects	Intrathecal morphine (ITM) showed similar effectiveness in pain treatment post knee replacement as SSFNB. However, ITM was associated with a higher incidence of cumbersome side effects, primarily nausea and pruritus

## Data Availability

Data available upon request.

## Appendix A

Database Search Terms. The table outlines the search terms employed across various databases (PubMed, MEDLINE, Embase, Web of Science, Cochrane Library, IEEE Xplore, Scopus) to explore postoperative pain management in orthopedic procedures. The search strings encompass keywords and phrases related to postoperative pain, orthopedic surgeries, and pain management strategies, facilitating comprehensive literature retrieval in the respective databases.

**Table:**

Database	Search Terms
PubMed	(“Postoperative Pain”[Mesh] OR postoperat* OR post-operat*) AND (“Orthopedic Procedures, Operative”[Mesh] OR orthoped* OR musculoskel* OR “extremity fracture*” OR fracture* OR limb OR limbs OR bone OR bones OR joint OR joints OR arthro* OR chondro* OR osteoto*) AND (“Pain Management”[Mesh] OR analgesi* OR anesthetic* OR anaesthetic* OR “pain control” OR “perioperative care” OR “postoperative care”) AND (technique* OR method* OR modalit* OR strateg* OR intervent* OR approach* OR manag* OR control*)
MEDLINE	Same as PubMed
Embase	‘postoperative pain’/exp OR postoperat* OR post-operat* AND ‘orthopedic procedure’/exp OR orthoped* OR musculoskel* OR ‘extremity fracture’ OR fracture* OR limb OR limbs OR bone OR bones OR joint OR joints OR arthro* OR chondro* OR osteoto* AND ‘pain management’/exp OR analgesi* OR anesthetic* OR anaesthetic* OR ‘pain control’ OR ‘perioperative care’ OR ‘postoperative care’ AND techniqu* OR method* OR modalit* OR strateg* OR intervent* OR approach* OR manag* OR control*
Web of Science	TS = (postoperative pain OR postoperat* OR post-operat*) AND TS = (orthopedic procedure* OR orthoped* OR musculoskel* OR “extremity fracture*” OR fracture* OR limb OR limbs OR bone OR bones OR joint OR joints OR arthro* OR chondro* OR osteoto*) AND TS = (pain management OR analgesi* OR anesthetic* OR anaesthetic* OR “pain control” OR “perioperative care” OR “postoperative care”) AND TS = (technique* OR method* OR modalit* OR strateg* OR intervent* OR approach* OR manag* OR control*)
Cochrane Library	postoperat* or post-operat* AND orthoped* or musculoskel* or “extremity fracture*” or fracture* or limb or limbs or bone or bones or joint or joints or arthro* or chondro* or osteoto* AND analgesi* or “pain management” or anesthetic* or anaesthetic* or “pain control” or “perioperative care” or “postoperative care” AND techniqu* or method* or modalit* or strateg* or intervent* or approach* or manag* or control*
IEEE Xplore	postoperat* or post-operat* AND orthoped* or musculoskel* or “extremity fracture*” or fracture* or limb or limbs or bone or bones or joint or joints or arthro* or chondro* or osteoto* AND analgesi* or “pain management” or anesthetic* or anaesthetic* or “pain control” or “perioperative care” or “postoperative care” AND techniqu* or method* or modalit* or strateg* or intervent* or approach* or manag* or control*
Scopus	postoperat* or post-operat* AND orthoped* or musculoskel* or “extremity fracture*” or fracture* or limb or limbs or bone or bones or joint or joints or arthro* or chondro* or osteoto* AND analgesi* or “pain management” or anesthetic* or anaesthetic* or “pain control” or “perioperative care” or “postoperative care” AND techniqu* or method* or modalit* or strateg* or intervent* or approach* or manag* or control*
